# Detection of 3D Cardiac metabolism after injection of hyperpolarized [1-13C]pyruvate

**DOI:** 10.1186/1532-429X-13-S1-M2

**Published:** 2011-02-02

**Authors:** Francesca Frijia, Florian Wiesinger, Maria Filomena Santarelli, Vincenzo Positano, Jan Henrik Ardenkjaer-Larsen, Rolf Schulte, Luca Menichetti, Giulio Giovannetti, Giacomo Bianchi, Giovanni Aquaro, Vincenzo Lionetti, Daniele De Marchi, Alessandra Flori, Luigi Landini, Fabio Anastasio Recchia, Massimo Lombardi

**Affiliations:** 1 Fondazione G.Monasterio CNR-Regione Toscana, Pisa, Italy; 2 GE Global Research, Munich, Germany; 3 Institute of Clinical Physiology, National Research Council, Pisa, Italy; 4 GE Healthcare, Huginsvej, 3400 Hillerod, Denmark; 5 Sector of Medicine, Scuola Superiore Sant'Anna, Pisa, Italy; 6 Scuola Superiore Sant'Anna, Pisa, Italy

## Introduction

MRI with hyperpolarised ^13^C represents a promising modality for *in-vivo* spectroscopy and provides a unique opportunity for *non-invasive* assessment of cardiac regional metabolism.

## Purpose

We present a method based on a volumetric IDEAL spiral CSI acquisition for obtaining spatial information on the metabolism of the whole heart after intravenous injection of hyperpolarized [1-13C]pyruvate in a large animal model with a clinical 3T scanner.

## Methods

Three healthy male mini-pigs (38±2 kg) were maintained under deep sedation; a dose of 20 mL of 230 mM [1-13C]pyruvate was administered over about 10 s by manual injection. Animal experiments were performed on a 3T GE Signa HDx scanner with a 13C quadrature birdcage coil.

[1-13C]pyruvate was polarized using a HyperSense DNP polariser with subsequent dissolution. The final injection solution contained 230 mM sodium [1-13C]pyruvate, 100 mM TRIS buffer, 0.27 mM Na_2_EDTA and 20 μM Dotarem with T≈37°C and pH ≈ 7.6.

Anatomical reference images were acquired in the axial plane with standard FIESTA sequence (body coil FOV=30x30 cm^2^, FA=20°, TE/TR=3.8ms/7.52ms, matrix 224x160, slice thickness 5 mm, 20 slices).

Metabolic information covering the heart were obtained using a 3D IDEAL spiral CSI prescribed on the same region imaged by the reference anatomical sequence (FOV= 30x30 cm, slab thickness=100mm) starting 20 seconds after the beginning of the hyperpolarized [1^13^C]-pyruvate injection. The IDEAL spiral CSI concept was implemented into a multi-slice, pulse-and-acquire sequence with a 2D spiral readout and phase encoding along the third dimension. A constant echo time shift of TE=0.9ms, 11 encoding steps and FA=7° were used to optimize the study for the considered frequencies.

The data was reconstructed using spectrally-preconditioned, minimum-norm CS inversion followed by gridding reconstruction implemented in Matlab. The reconstruction on cardiac short axis (SA) and image fusion was performed by PMOD software.

## Results

Pyruvate and its metabolic products lactate and bicarbonate were detected in the heart. Metabolic maps overlaid on anatomical images are shown in Figure 1. On SA sections the metabolites signal resulted correctly localized in cardiac structures: pyruvate more evident in ventricular cavity, bicarbonate in myocardial wall.

## Conclusions

This study demonstrated that the volumetric spatial localization of ^13^C metabolites on the whole pig heart can be acquired with a 3D IDEAL spiral CSI sequence. This allows investigation of the metabolic behaviour over all the heart, both in normal and pathological conditions.

